# Selective manipulation of electronically excited states through strong light–matter interactions

**DOI:** 10.1038/s41467-018-04736-1

**Published:** 2018-06-11

**Authors:** Kati Stranius, Manuel Hertzog, Karl Börjesson

**Affiliations:** Department of Chemistry and Molecular Biology, University of Gothenburg, Kemigården 4, 412 96 Gothenburg, Sweden

**Keywords:** Excited states, Fluorescence spectroscopy, Organic molecules in materials science

## Abstract

Strong coupling between light and matter leads to the spontaneous formation of hybrid light–matter states, having different energies than the uncoupled states. This opens up for new ways of modifying the energy landscape of molecules without changing their atoms or structure. Heavy metal-free organic light emitting diodes (OLED) use reversed intersystem crossing (RISC) to harvest light from excited triplet states. This is a slow process, thus increasing the rate of RISC could potentially enhance OLED performance. Here we demonstrate selective coupling of the excited singlet state of Erythrosine B without perturbing the energy level of a nearby triplet state. The coupling reduces the triplet–singlet energy gap, leading to a four-time enhancement of the triplet decay rate, most likely due to an enhanced rate of RISC. Furthermore, we anticipate that strong coupling can be used to create energy-inverted molecular systems having a singlet ground and lowest excited state.

## Abstract (web summary)

Manipulating energy levels in molecules could allow applications such as improving organic LEDs. Here, the authors show evidence that reversed intersystem crossing can be enhanced in Erythrosine B coupled to a cavity by selectively manipulating the energy of the singlet state.

## Introduction

Chemists can today synthesize virtually any molecule imaginable. By fine-tuning the molecular structure, optimized physical, chemical, or biological properties are routinely achieved. However, even though molecular optimization has reshaped the world we live in, there is always a point at which the laws of physics limits the performance of molecular systems. Tailoring of molecular properties can be achieved through strong coupling between molecular states and the zero-point fluctuations of the electromagnetic field (vacuum field)^1–4^. The formed hybrid states (exciton polaritons) have unique chemical and physical properties and can be viewed as a linear combination of light (vacuum field) and matter (molecules). Strong coupling between organic molecules and light has gathered considerable attention in the past couple of years due to the vast number of possible applications it offers in physical and chemical sciences^5^. For example, strong coupling has been observed in molecular crystals^6–8^, J-aggregates^9–13^, polymers^14–16^, small molecules in a polymer or silicon dioxide matrix^17–26^, large light-harvesting complexes^27^ and even liquid crystals^28^. Furthermore, it has been shown to change chemical reactivity^29^, work function^23^, and phase transitions^30^; increase electronic conductivity^31^ and energy transfer, and modify ground-state thermodynamics^22^ and reaction-energy landscapes^20^. Strong coupling between single molecules and a vacuum field has now also been realized^32^.

**Fig. 1:**
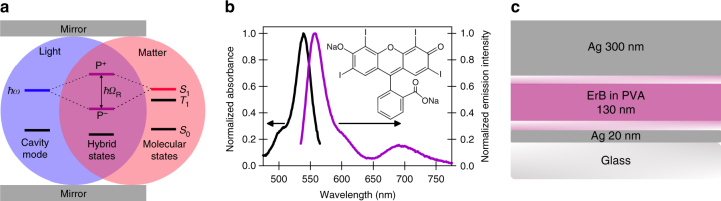
Molecular system for realizing strong light–matter coupling. **a** Jablonski diagram presenting strong coupling of a molecular state (*S*_1_) with a cavity mode (*ħω*) that has the same energy and leads to the formation of two new hybrid light–matter states, *P*^+^ and *P*^−^, which are separated in energy by the Rabi splitting (*ħΩ*_R_). **b** Absorption (black line) and emission (purple line) spectra of 1 wt% ErB in a PVA matrix deposited on a glass substrate. Inset shows the chemical structure of ErB. **c** Structure of the non-transparent Fabry–Pérot cavity, where two Ag mirrors sandwich a PVA film containing ErB. PMMA 10 nm films, deposited between the mirror and chromophore layer, were used to prevent direct contact between ErB and the Ag mirror

Optical cavities, e.g., two parallel mirrors, can be used to couple light and matter. The coupling leads to the formation of two new optically allowed hybrid light–matter states, *P*^−^ and *P*^+^, which are separated in energy by the Rabi splitting (*ħΩ*_R_; Fig. 1a). Using the rotating wave approximation, in the limit of large number of molecules (*N*), the Hamiltonian of the system can be approximated by^2^:

1 $$\begin{array}{l}{\hat {\cal{H}}} = {\hat {\cal{H}}}_{\mathrm{mol}} + {\hat {\cal{H}}}_{\mathrm{cav}} + {\hat {\cal{H}}}_{\mathop{\rm{int}}} \approx \hbar \omega _0\left( { - \frac{N}{2} + \hat b^\dagger \hat b} \right)\\ + \hbar \omega \,\hat a^\dagger \hat a + \hbar g\sqrt N \left( {\hat a^\dagger \hat b + h.c.} \right)\end{array}$$

where, $g = - {\mathbf{d}}{\cal{E}}{\mathrm{/}}\hbar$ is the coupling constant between the cavity mode and the electronic transition of the molecule, **d** is the respective transition dipole moment, and *ε* is the vacuum electric field. It should be noted that *ε* is finite even in the absence of an external electromagnetic field. This is due to vacuum fluctuations inside the cavity and all measurements and considerations here is made in this low photon regime. Solving the eigenmodes of Eq. 1 gives the magnitude of the vacuum Rabi splitting:

2 $$\hbar {{\varOmega }}_{\mathrm{R}} = 2g\sqrt N$$

The magnitude of the Rabi splitting is thus proportional to the transition dipole moment associated with the state being hybridized and the square root of the concentration of the molecule inside the cavity.

The linear dependence of the Rabi splitting on the magnitude of the transition dipole moment (Eq. 2) has profound practical and theoretical implications. The electronic ground state of an organic molecule is of singlet character. Furthermore, Hund’s rule states that triplet states always are lower in energy than their corresponding singlet states. Thus the first excited state of an organic molecule is a triplet state (dark state), having a low transition dipole moment to the ground state. The inefficient emission of light from triplet states is problematic in the field of light-emitting organic electronics. To address this, there has been a considerable activity in the research field of thermally activated delayed fluorescence, in which molecules are engineered to have as small a triplet–singlet energy difference as possible, enabling thermal excitation of the singlet state from triplet states^33^. This concept has been relatively successful, but still, the rate of emission from a singlet state is several orders of magnitude faster compared to the rate of delayed fluorescence^34^. However, the dependence of the Rabi splitting on the magnitude of the transition dipole moment suggests that strong coupling could be used to selectively modify the energy levels of singlet states, without significantly perturbing the energy levels of triplet states. This would enable decreasing the energy difference between hybrid states of singlet character and triplet states, possibly leading to an enhanced rate of thermally activated reversed intersystem crossing (RISC) and potentially enabling the use of conventional fluorophores (e.g., fluorescein and rhodamine) in efficient organic light-emitting devices.

Here we demonstrate on the possibility to selectively tune excited state processes by modifying the energy landscape through coupling with the vacuum field. We start by showing that it is possible to couple a singlet state to the vacuum field without perturbing the energy level of a nearby triplet (dark) state. Using time-resolved spectroscopy, we continue with showing that the triplet decay rate is four times increased in the strong coupling regime, most likely due to an enhanced rate of RISC. Finally, by observing the temperature dependence of the triplet decay rate, the lowering of the activation energy of RISC is in good agreement with the energy difference between the excitonic and lower polaritonic state, as measured with absorbance spectroscopy.

## Results

### Formation of hybrid light–matter states of Erythrosine B

We chose Erythrosine B (ErB) as a model compound for observing the dynamics of excited singlet and triplet states. ErB is the tetra-iodized derivative of fluorescein (Fig. 1b) and when dissolved in a rigid polymer matrix, it simultaneously exhibits both fluorescence and phosphorescence (Fig. 1b). The absorbance spectrum shows, apart from a small shoulder, a single transition centered at 538 nm, with an oscillator strength of 0.5^35^. The fluorescence is centered at 558 nm and is a mirror image to the absorbance, and the phosphorescence is relatively broad and structureless with a maximum at 691 nm. Thus information on the energy levels of the lowest excited singlet and triplet states are realized in a single experiment at room temperature. Furthermore, ErB exhibits e-type delayed fluorescence^36^, i.e., fluorescence arising from thermal population of the singlet state from a triplet state, which enables straightforward monitoring of singlet/triplet dynamics through time-resolved spectroscopy.

**Fig. 2:**
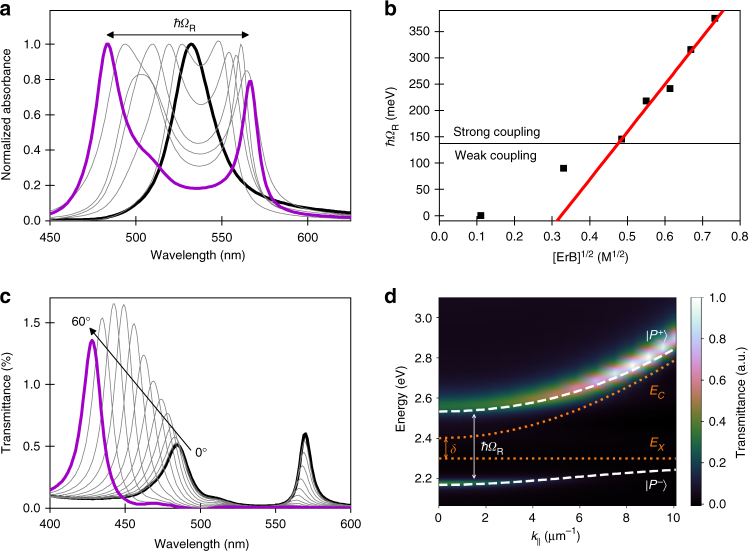
Characterization of hybrid light–matter states. **a** Absorbance spectra of ErB/PVA films inside a cavity that has an ErB concentration between 0.01 M (black) and 0.54 M (purple). The thickness of the cavities were optimized as to give a roughly equal magnitude of the lower and upper polariton absorption. **b** The linear dependence of the Rabi splitting in the strong coupling regime on the square root of [ErB]. The strong/weak coupling limit is visualized as a line at 140 meV. **c** Transmission spectra of a 0.54 M ErB/PVA film inside a cavity having two semitransparent mirrors as a function of incidence angle. **d** Dispersion plot constructed from the data in (**c**) (color map) overlaid with fitted polariton dispersion (white dashed line). The uncoupled non-dispersive ErB absorption (*E*_x_) and cavity dispersion (*E*_c_) are shown as dashed lines. The Rabi splitting (*ħΩ*_R_) is the energy gap between *P*^−^ and *P*^+^ at the intersection (or minimum gap) of *E*_x_ and *E*_c_

To probe light–matter interactions, ErB was inserted into a Fabry–Pérot cavity (Fig. 1c). The strong coupling regime is characterized by the cavity mode splitting into two new bands, *P*^−^ and *P*^+^, separated by the Rabi splitting (Fig. 2a). Strong coupling is achieved when the magnitude of the Rabi splitting is larger than the full width at half maximum (FWHM) of both the ErB absorbance band and the cavity mode, which occurred when the Rabi splitting was >140 meV. Entrance to the strong coupling regime can be also seen in Fig. 2b where the linear dependence of the Rabi splitting on the square root of [ErB] is seen in the strong coupling regime (Eq. 2). The hybrid states are dispersive by nature, and thus they have properties that are different from those of the individual molecular states or cavity modes (Fig. 2c, d). With an increasing angle of incidence, the absorbance spectra show a hypsochromic shift together with a reduction of *P*^−^ and increment of *P*^+^ absorption. Transfer matrix calculations confirmed the experimental observations (Supplementary Fig. 1). From the Hopfield coefficients that quantify the exciton and photon ratio in each of the polariton branches (Supplementary Fig. 2), we observed that, at normal incidence angle, both the molecular and optical modes contribute roughly equally to the hybrid states. However, at larger *k*_||_ values, *P*^+^ has increasing photonic contribution following the cavity dispersion (Eq. 7 in Methods) and P^−^ has increasing material contribution following the excitonic transition energy. Thus all optical measurements from here on were recorded normal to the cavity surface.

### Lowering of the triplet–singlet energy gap

**Fig. 3:**
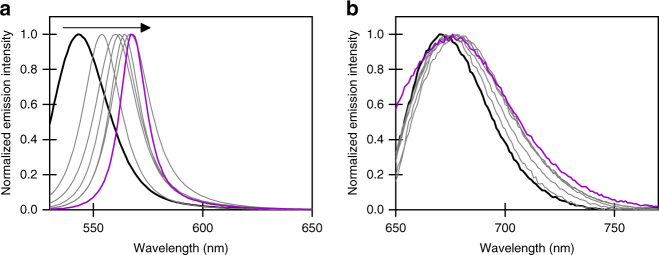
Selective coupling of excited states and decrease of triplet–singlet energy gap. **a** Fluorescence/*P*^−^ emission and **b** phosphorescence spectra of ErB/PVA films inside a cavity with an ErB concentration between 0.01 (black) and 0.54 M (purple), excited at the maximum of the *P*^+^ absorbance. The clean phosphorescence band was obtained by subtracting the fluorescence tail from the phosphorescence spectra (Supplementary Fig. 3)

By making use of the optimal conditions ascertained above, we were able to set up and study the difference in energy between the triplet and hybrid singlet states. The cavity containing 0.54 M ErB displays the largest Rabi splitting (375 meV; Fig. 2a), which would correspond to a lowering of the *P*^−^- state energy by 187.5 meV compared to the uncoupled singlet-state energy. However, owing to asymmetry of the ErB absorbance (Fig. 1b), the splitting was not symmetric and the lower polaritonic band *P*^−^ was actually shifted by 135 meV compared to the uncoupled state. The lowering of the *P*^−^state energy can also be followed by the emission arising from the *P*^−^ state (Fig. 3a). Fluorescence arising from uncoupled ErB has a maximum at 543 nm (Supplementary Table 1). As the strong coupling regime is reached through an increase in ErB concentration, the *P*^−^ emission is gradually red-shifted. The emission maximum of the cavity containing 0.54 M of ErB is shifted by 24 nm (96 meV) compared to uncoupled ErB fluorescence (Supplementary Table 1, Supplementary Note 1). In contrast, the phosphorescence band inside the cavity was not red-shifted compared to reference samples when the concentration of ErB was increased (Fig. 3b, Supplementary Table 1 and Supplementary Fig. 3). The lack of concentration dependence of phosphorescence shows that it is possible to couple the singlet state to the vacuum field without significantly perturbing the corresponding triplet states. Thus, with an increase in the coupling strength of the singlet (polaritonic) state, a simultaneous reduction of the energy difference between the triplet and singlet (polaritonic) state (Δ*E*_TS_) is achieved.

To show that emission occurs as a consequence of excitation to the polaritonic states, rather than to uncoupled molecules, excitation spectra of cavities with different ErB concentrations were monitored at both the lower polariton and the phosphorescence emission maxima (Supplementary Fig. 4). The excitation spectra are in reasonable agreement (Supplementary Note 2) with corresponding absorbance spectra for all cavities at both emission wavelengths. Thus relaxation from the upper and lower polariton states to the triplet state is equally efficient. In addition, the presence of polaritonic peaks in the excitation spectra monitored at the phosphorescence maxima proofs that the polaritons (or excitation reservoir) can undergo ISC as reported earlier^37^. Furthermore, no emission can be seen when exciting at wavelengths where uncoupled ErB absorb light.

**Fig. 4:**
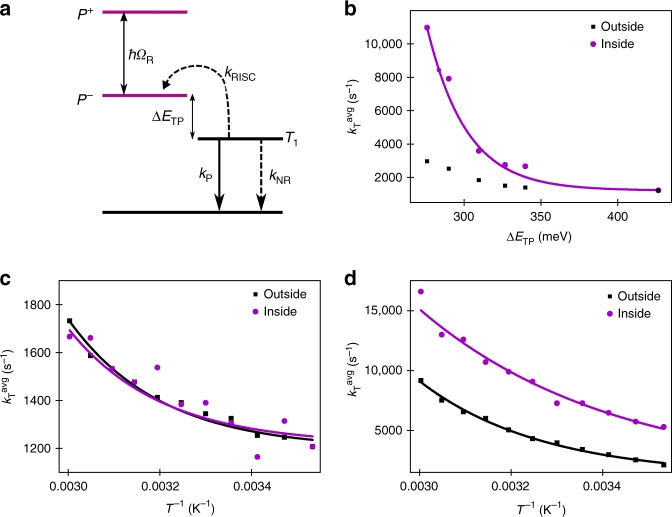
Increased rate of reversed intersystem crossing. **a** The energy diagram describing the kinetics of the triplet-state depopulation pathways inside a cavity. *k*_P_, *k*_NR_, and *k*_RISC_ are the rates of phosphorescence, non-radiative decay, and reverse intersystem crossing, respectively. Δ*E*_TP_ is the energy difference between *T*_1_ and *P*^−^. **b** The increase of the average total and the fitted rate constant (Eq. 3) of the triplet-state depopulation (*k*^avg^_T_) inside the cavity as a function of the energy difference between *T*_1_ and *P*^−^ (Δ*E*_TP_*)*. The rate constants outside the cavity with equivalent concentration are shown for comparison. **c**, **d** Temperature dependence of the average total rate constant of the triplet-state depopulation (*k*^avg^_T_) and exponential fit (Eq. 3) for 0.02 M (**c**) and 0.54 M (**d**) ErB/PVA films outside and inside a cavity. Samples were excited at the maximum of the *P*^+^ absorbance peak and monitored at 690 nm

Since the triplet–state energy is unaffected in the cavity, we assumed that the triplet–singlet (polaritonic) energy gap ($\Delta E_{{\mathrm{TP}}}$) decreases with the Rabi splitting. At low concentration, $\Delta E_{{\mathrm{TS}}}$ inside the cavity was 436 meV and it was determined from the spectral separation of the maxima of the fluorescence and phosphorescence (Supplementary Table 1). The obtained value agrees well with the previously reported value for ErB in a polyvinyl alcohol (PVA) matrix (Supplementary Note 3)^36^. Figure 4a represents the energy diagram that describes the kinetics of the triplet-state depopulation pathways after the triplet state is populated by ISC from *S*_1_/*P*^−^ to *T*_1_. The triplet state can be assumed to be populated instantly since ISC from the initially excited *S*_1_/*P*^−^ state to *T*_1_ is much faster (nanoseconds) than the deactivation rates (microseconds) of *T*_1_. The phosphorescence lifetime is given by:

3 $$\tau _{\mathrm{T}}^{ - 1} = k_{\mathrm{T}} = k_{\mathrm{P}} + k_{{\mathrm{NR}}} + k_{{\mathrm{RISC}}} = k_{\mathrm{P}} + k_{{\mathrm{NR}}} + k_{{\mathrm{ISC}}}{\mathrm{exp}}\left( { - \frac{{\Delta E_{{\mathrm{TS}}/{\mathrm{P}}}}}{{RT}}} \right)$$

where *k*_T_ is the total rate constant of the triplet state, *k*_P_ is the radiative rate of phosphorescence, *k*_NR_ is the non-radiative decay rate, and *k*_RISC_ and *k*_ISC_ are the rates of RISC from *T*_1_ to *S*_1_/*P*^−^ and ISC from *S*_1_/*P*^−^ to *T*_1_, respectively. The probability that the molecule will overcome the energy barrier between the triplet state and the first excited singlet/polaritonic state is given by the Boltzmann distribution factor, exp(–Δ*E*_TS/P_*/RT*)^36^.

### Increased RISC due to strong coupling

To determine how the lowering of Δ*E*_TP_ affects the excited state dynamics of ErB, time-resolved phosphorescence decays were measured. The triplet state was monitored since it does not show any coupling to the vacuum field and thus remains stationary in energy in the strong coupling regime. The phosphorescence lifetimes (*τ*_T_) were obtained from the measured phosphorescence decays by using stretched-exponential tail-fits (Supplementary Note 4). The phosphorescence decays and fitting results for all uncoupled and coupled samples are shown in Supplementary Fig. 5 and Supplementary Table 2, respectively. Figure 4b shows the average total rate constant of the triplet state (*k*^avg^_T_) depopulation as the energy difference between *T*_1_ and *P*^−^ (Δ*E*_TP_) is decreasing (Supplementary Note 5, Supplementary Fig. 6). At low concentrations, when the system is not in the strong coupling regime, the rate of triplet decay is the same inside the cavity as compared to a bare film, thus indicating that the presence of the cavity does not induce any simple electromagnetic effects (such as the Purcell effect). However, at high concentrations of ErB (thus in the strong coupling regime), the phosphorescence decay was four times less inside the cavity as compared to outside. Furthermore, the change of *k*_T_ can successfully be reproduced by Eq. 3 (Supplementary Table 3), indicating that the change in *k*_T_ is due to a reduced triplet-polaritonic energy gap. Importantly, the photophysical pathways of triplet-state deactivation are modified even though only the singlet state was coupled to the vacuum field.

To further strengthen the hypothesis that the increase in the rate constant of triplet-state deactivation is due to a lowering of *ΔE*_TP_, the temperature dependence of the phosphorescence lifetime for uncoupled (0.01 M) and strongly coupled (0.54 M) ErB was determined (Fig. 4c, d, respectively; see Supplementary Fig. 7 and Supplementary Table 5–6 for raw data). The temperature dependence of the phosphorescence rate constants of uncoupled ErB (0.01 M) inside the cavity overlaps well with the values outside a resonant cavity (Fig. 4c). However, the observed temperature dependence of the phosphorescence rate constants of ErB at high concentration (0.54 M) inside the cavity (strongly coupled) differs from those outside a resonant cavity (Fig. 4d). To estimate the change of the triplet–singlet/polaritonic energy gap inside the cavity, Eq. 3 was used to fit the temperature dependence of the phosphorescence (Supplementary Table 6). The obtained Δ*E*_TS/P_ values were 322 meV and 204 meV for ErB at 0.54 M outside and inside the cavity, respectively. This corresponds to a lowering of the triplet–singlet/polaritonic energy gap by 118 meV. Although the Δ*E*_TS/P_ values are slightly smaller than the values obtained from steady-state analysis, the lowering of the energy gap agrees well with the shift in the lower polaritonic absorption band compared to the uncoupled state (Supplementary Table 7). This supports the hypothesis that the decrease of the phosphorescence lifetime is due to an increased rate of RISC caused by a lowering of the triplet–singlet/polaritonic energy gap. Interestingly, in the temperature-dependence analysis, a reduction of the fitted *k*_ISC_ was noticed. Thus the coupling between the triplet and polaritonic states seems to be slightly lower as compared to the coupling between the triplet and singlet states. The lower coupling works against the lowering of Δ*E*_TS/P_ and the effect is thus unfavorable in singlet-harvesting applications. However, it is too early to say if a lower coupling between triplet and polaritonic states is system specific or a general feature for organic molecules in the strong coupling regime.

## Discussion

We have successfully coupled the singlet excited state of ErB to the vacuum field, without perturbing the energy of the corresponding triplet state. By doing so, the triplet–singlet/polaritonic energy gap was reduced, resulting in an increased triplet decay rate, presumably due to an increased rate of RISC. We envisage that the described concept could be used to lower the energy of excited singlet states under that of the corresponding triplet states. This would allow for molecular systems to have a ground and first excited state of singlet character and lead to numerous practical applications in applied fields, such as light-emitting organic electronics.

## Methods

### Cavity preparation

The Fabry–Pérot cavities were built on glass substrates (25 × 25 mm^2^), which were precleaned by sonication for 15 min in alkaline solution (0.5% of Hellmanex in distilled water), and then rinsed with water and sonicated for 1 h in water and ethanol, respectively. The cleaned glass substrates were dried in an oven overnight before cavity preparation. Polymethyl methacrylate (PMMA, Sigma Aldrich) was dissolved in toluene (5 mg mL^−1^). ErB (Sigma Aldrich) was dissolved in water containing PVA (88% hydrolyzed, Acros Organics, 25–35 mg mL^−1^). Polymer films were deposited by spin-coating (Laurell Technologies WS-650), at speeds optimized to give roughly 10 nm PMMA and 130 nm PVA/ErB films. Thickness of the polymer films were measured using profilometry (KLA Tencor D-100). Ag mirrors were fabricated by vacuum sputtering deposition (HEX, Korvus Technologies). A semitransparent 20 nm Ag film was sputtered on top of the glass plate and the thick 300 nm films was sputtered on top of the polymer layers to form a sealed cavity that could be stored in nitrogen atmosphere for months without there being any effects of Ag oxidation on the cavity properties. PMMA 10 nm films were used to prevent direct contact between ErB and the Ag mirrors (direct contact could affect both radiative and non-radiative rate constants of ErB). The thickness of the PVA/ErB film was optimized to give the *λ*/2 cavity mode in resonance with the absorbance maximum of ErB at 538 nm.

To determine the absorbance of the cavity, we used one semitransparent (20 nm) and one non-transparent (300 nm) mirror. The absorbance was calculated ($A = \mathrm{log}\frac{1}{R}$) by assuming that transmission and scattering by the cavity is zero and probed by using reflectance spectroscopy. The total concentration of ErB inside the cavity was calculated from the absorbance spectra of reference samples with only one Ag mirror. The quality factor (*Q* = *λ*_r_/Δ*λ*) of empty cavities was around 15, which is in line with the FWHM of the absorbance of ErB. For dispersion measurements, transparent cavities were prepared so that both mirrors were 50 nm thick. Reference samples were prepared using same parameters and solutions for spin-coating the polymer layers and sputtering 300 nm Ag layer on top.

In addition, because of the low intensity of the phosphorescence from the cavity the lifetime measurements were done for cavities with slightly thinner Ag mirror (18 nm, *Q* = 18), giving a substantial higher signal-to-noise ratio. The absorbance and emission data for these cavities are presented in Supplementary Fig. 8–9.

### Optical measurements

Steady-state transmission and reflectance spectra were measured using a spectrophotometer equipped with a small-angle specular reflectance accessory (Lambda 650, Perkin Elmer). Reflectance spectra were measured relative to a standard reflectance mirror and the angular transmittance was measured using a horizontal Glan-Taylor polarizer. Steady-state emission spectra were measured with a spectrofluorometer (Spex Fluorolog, JY Horiba or FLS1000, Edinburgh Instruments). The optical properties of the nontransparent cavities were studied through the glass in front-face geometry.

Direct time-resolved emission decay data were recorded using a nanosecond Nd:YAG Surlite pulsed laser with a repetition frequency of 10 Hz and an FWHM pulse of 7 ns. The desired excitation wavelength was obtained using a Surlite OPO (Continuum). Phosphorescence was detected using a five-stage photomultiplier (Applied Photophysics) and recorded with an oscilloscope interfaced with a custom-made LabVIEW program. The decays were recorded using excitation pulse energy <0.2 mJ cm^−2^. During the temperature-dependent measurements, the excitation spot was changed a few times to avoid degradation of the sample throughout the duration of the experiments.

### Stretched exponential fitting

Phosphorescence decays were analyzed using a stretched exponential function^38^:

4 $$I\left( t \right) = I\left( 0 \right){\mathrm{exp}}\left[ { - \left( {t{\mathrm{/}}\tau } \right)^\beta } \right]$$

where *I*(0) is the initial amplitude, *τ* is the time constant characterizing the position, and *β* is width of the distribution of decay times. *β* varies from 0 to 1 and quantifies the non-exponential nature of the decay.

In order to compare the distribution of phosphorescence lifetimes, the average phosphorescence time constants were calculated^38^:

5 $$\tau ^{{\mathrm{avg}}} = \tau { {\varGamma }}\left( {1 + \frac{1}{\beta }} \right)$$

where *Γ* is the Gamma function.

### Simulating the angle-resolved reflectivity measurements

To model the angle-resolved reflectivity measurements, we modeled the system as a coupled oscillator. Inside a Fabry–Pérot cavity, we can model the coupling between an exciton and a photon using the following Hamiltonian^39,40^:

6 $$\hat H\left( {k_\parallel } \right) = \left( {\begin{array}{*{20}{c}} {E_{\mathrm{X}}\left( {k_\parallel } \right) - i\hbar { {\varGamma }}_{\mathrm{X}}} & {V_{\mathrm{A}}} \\ {V_{\mathrm{A}}} & {E_{\mathrm{C}}\left( {k_\parallel } \right) - i\hbar { {\varGamma }}_{\mathrm{C}}} \end{array}} \right)$$

where *E*_X_ is the excitonic transition energy, and *ħΓ*_X_ and ħ*Γ*_C_ are the broadening of the excitonic transition and the cavity (FWHM). *E*_C_ is the energy dispersion of the cavity given by^39,40^:

7 $$E_{\mathrm{C}}\left( \theta \right) = \frac{{E_0}}{{\sqrt {1 - \left( {\frac{{\sin \left( \theta \right)}}{{n_{\mathrm{{eff}}}}}} \right)^2} }}$$

where *E*_0_ _*=*_ *E*_X_−*δ* with *δ* as the cavity detuning (*δ* = 100 meV), *n*_eff_ is the refractive index inside the cavity (PVA matrix, *n*_eff_ = 1.49), and *θ* is the angle of incidence. *V*_A_ is the interaction potential representing the coupling of the two oscillators related to the Rabi splitting *ħΩ*_R_, when *E*_C_ *=* *E*_*X*_ and is given by:

8 $$V_{\mathrm{A}} = \frac{1}{2}\sqrt {\left( {\hbar { {\varOmega }}_{\mathrm{R}}} \right)^2 + \left( {\hbar { {\varGamma }}_{\mathrm{C}} - \hbar { {\varGamma }}_{\mathrm{X}}} \right)^2}$$

Eigenvalues of *Ĥ* give the energy of the upper and lower polariton branch (*P*^+^ and *P*^−^ respectively) with *|P*^+^> and *|P*^−^> as eigenvectors:

9 $$E^{P^ + /P^ - } = \frac{1}{2}\left[ {E_{\mathrm{X}} + E_{\mathrm{C}} - i\left( {\hbar { {\varGamma }}_{\mathrm{X}} - \hbar { {\varGamma }}_{\mathrm{C}}} \right)} \right] \pm \sqrt {V_A^2 + \frac{1}{4}\left( {E_{\mathrm{X}} - E_{\mathrm{C}} - i\left( {\hbar { {\varGamma }}_{\mathrm{C}} - \hbar { {\varGamma }}_{\mathrm{X}}} \right)} \right)^2}$$

### Data availability

Data are available on request from the authors.

## Electronic supplementary material

Supplementary Information
