# Publisher Correction: Selective manipulation of electronically excited states through strong light–matter interactions

**DOI:** 10.1038/s41467-020-19359-8

**Published:** 2020-10-27

**Authors:** Kati Stranius, Manuel Hertzog, Karl Börjesson

**Affiliations:** Department of Chemistry and Molecular Biology, University of Gothenburg, Kemigården 4, 412 96 Gothenburg, Sweden

**Keywords:** Excited states, Fluorescence spectroscopy, Organic molecules in materials science

Correction to: *Nature Communications* 10.1038/s41467-018-04736-1, published online 11 June 2018.

The original PDF version of this Article contained errors in Eqs. 2, 5, 6, 8 and 9, which were missing all the Ω and Γ terms, and incorrectly read:

$${\hbar} _R = 2g\sqrt N$$(2)

$$\tau ^{{\mathrm{avg}}} = \tau \left( {1 + \frac{1}{\beta }} \right)$$(5)

$$\hat H\left( {k_\parallel } \right) = \left( {\begin{array}{*{20}{c}} {E_{\mathrm{X}}\left( {k_\parallel } \right) - i{\hbar} _{\mathrm{X}}} & {V_{\mathrm{A}}} \\ {V_{\mathrm{A}}} & {E_{\mathrm{C}}\left( {k_\parallel } \right) - i{\hbar} _{\mathrm{C}}} \end{array}} \right)$$(6)

$$V_{\mathrm{A}} = \frac{1}{2}\sqrt {\left( {{\hbar} _R} \right)^2 + \left( {{\hbar} _{\mathrm{C}} - {\hbar} _{\mathrm{X}}} \right)^2}$$(8)

$$E^{P^ + /P^ - } = \frac{1}{2}\left[ {E_{\mathrm{X}} + E_{\mathrm{C}} - i\left( {{\hbar} _{\mathrm{X}} - {\hbar} _{\mathrm{C}}} \right)} \right] \pm \sqrt {V_A^2 + \frac{1}{4}\left( {E_{\mathrm{X}} - E_{\mathrm{C}} - i\left( {{\hbar} _{\mathrm{C}} - {\hbar} _{\mathrm{X}}} \right)} \right)^2}$$(9)

The correct forms of Eqs. 2, 5, 6, 8 and 9 are:

2 $${\hbar}{\it{\Omega}}_R = 2g\sqrt N$$

5 $$\tau ^{{\mathrm{avg}}} = \tau {\it{\Gamma}}\left( {1 + \frac{1}{\beta }} \right)$$

6 $$\hat H\left( {k_\parallel } \right) = \left( {\begin{array}{*{20}{c}} {E_{\mathrm{X}}\left( {k_\parallel } \right) - i{\hbar} {\it{\Gamma}}_{\mathrm{X}}} & {V_{\mathrm{A}}} \\ {V_{\mathrm{A}}} & {E_{\mathrm{C}}\left( {k_\parallel } \right) - i{\hbar} {\it{\Gamma}}_{\mathrm{C}}} \end{array}} \right)$$

8 $$V_{\mathrm{A}} = \frac{1}{2}\sqrt {\left( {{\hbar} {\it{\Omega} }_{\mathrm{R}}} \right)^2 + \left( {{\hbar} {{\it{\Gamma} }}_{\mathrm{C}} - {\hbar} {{\it{\Gamma} }}_{\mathrm{X}}} \right)^2}$$

9 $$\begin{array}{ccccc}\\ E^{P^ + /P^ - } = &\frac{1}{2}\left[ {E_{\mathrm{X}} + E_{\mathrm{C}} - i\left( {{\hbar} {\it{\Gamma}}_{\mathrm{X}} - {\hbar} {\it{\Gamma} }_{\mathrm{C}}} \right)} \right]\pm \sqrt {V_A^2 + \frac{1}{4}\left( {E_{\mathrm{X}} - E_{\mathrm{C}} - i\left( {{\hbar} {{\it{\Gamma}}}_{\mathrm{C}} - {\hbar} {\it{\Gamma}}_{\mathrm{X}}} \right)} \right)^2} \\ \end{array}$$

The pdf has been updated to include these corrections. The original HTML version was correct and has not been changed.

