# Supplementary material for: Selective manipulation of electronically excited states through strong light–matter interactions
Source: Nat Commun. 2018 Jun 11;9:2273. doi: 10.1038/s41467-018-04736-1 (PMC5995866; doi:10.1038/s41467-018-04736-1)
Supplement: Supplementary file 1 — Supplementary Information [file 41467_2018_4736_MOESM1_ESM.pdf]

Supplementary Information: Selective Manipulation of Electronically Excited States through Strong Light–Matter Interactions

Stranius et al.

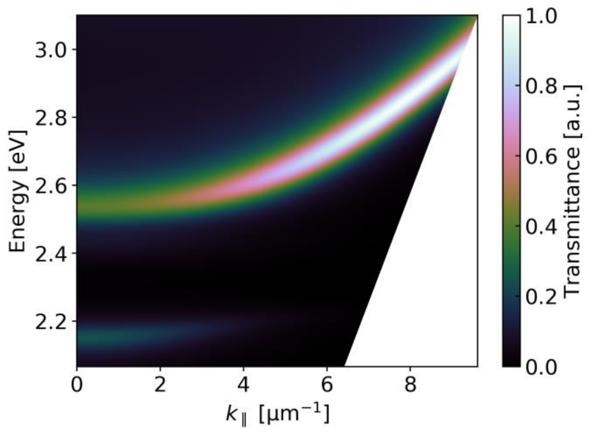

Supplementary Figure 1: Simulated angle dependent Transmission. Modelling was done for a 0.54 M ErB/PVA film inside a transparent cavity, using the Transfer matrix method[1].

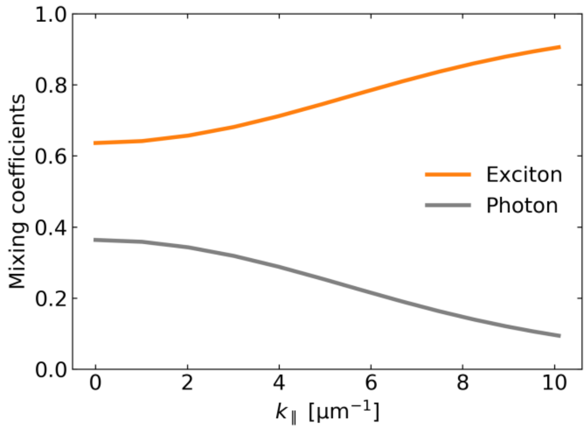

Supplementary Figure 2: Hopfield coefficients for P^+ and P^- Coefficients were calculated using the coupled oscillator model for a 0.54 M ErB/PVA film inside a cavity.

Supplementary Table 1: Changes in steady-state absorption and emission spectra due to strong-coupling. The S_1 absorption (λ_{S1}^{abs}) and fluorescence (λ_{S1}^{emis}) outside a cavity, P- absorption (λ_{P1}^{abs}) and fluorescence (λ_{P1}^{emis}) inside a cavity, and phosphorescence maxima (λ_{T1}^{emis}) outside and inside a cavity at different concentrations of ErB in PVA.

[ErB] (M)		$\lambda_{S1/P1}^{abs}$ (nm)	$\lambda_{S1/P1}^{emis}$ (nm)	λ_{T1}^{emis} (nm)
0.01	outside	538	555	690
	inside	538	543	672
0.11	outside	538	561	692
	inside	548	554	675
0.24	outside	538	564	693
	inside	554	560	674
0.30	outside	537	565	695
	inside	558	562	677
0.38	outside	536	565	696
	inside	561	564	676
0.45	outside	536	566	695
	inside	565	567	679
0.54	outside	534	569	695
	inside	567	567	675

Supplementary Note 1: Comments on the effect of the cavity and concentrations used.

The resonant cavity acts as a spectral filter, which means that the emission spectrum is filtered by the transmission properties of the cavity. Compared to the values outside the cavity, the fluorescence and phosphorescence maxima of the uncoupled ErB (0.01 M) inside the cavity are shifted to the blue by 12 and 18 nm, respectively (Supplementary Table 1). Since a shift in the whole emission spectra was observed even at low concentrations (uncoupled samples), it was assigned to be caused by the filtering effect. Due to this filter effect, the cavity transmission overlapped the fluorescence in higher concentration cavities; the filtering property of the cavity was assigned to cause the smaller shift of the fluorescence band as compared to the shift in P^- absorbance. In addition, the out-coupling efficiency of the cavities was decreased dramatically in the phosphorescence region of the electromagnetic spectra (650–750 nm). Thus, due to the low intensity, the phosphorescence region was measured separately using larger slit sizes (Supplementary Fig. 3. By subtracting the fluorescence tail from the phosphorescence spectra, the clean phosphorescence band was obtained (Fig. 4b).

Furthermore, the concentration of ErB required to reach the strong coupling regime is quite high. At high concentrations, ErB is known to aggregate[2-4] resulting in red-shifted emission (Supplementary Table 1) and fluorescence quenching, which was observed as a decrease of the overall emission intensity at high concentrations. Therefore, the emission from the cavities containing even higher concentrations than reported here was not any more measurable due to low S/N ratio.

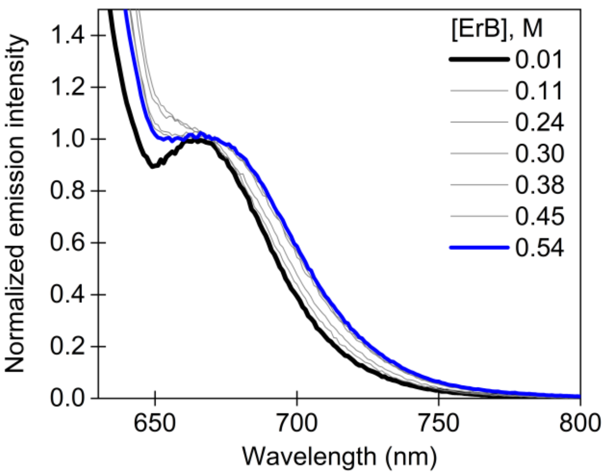

Supplementary Figure 3: Phosphorescence measured from inside a cavity. Phosphorescence spectra normalized at phosphorescence maxima for ErB/PVA films inside a cavity at different concentrations of ErB in PVA

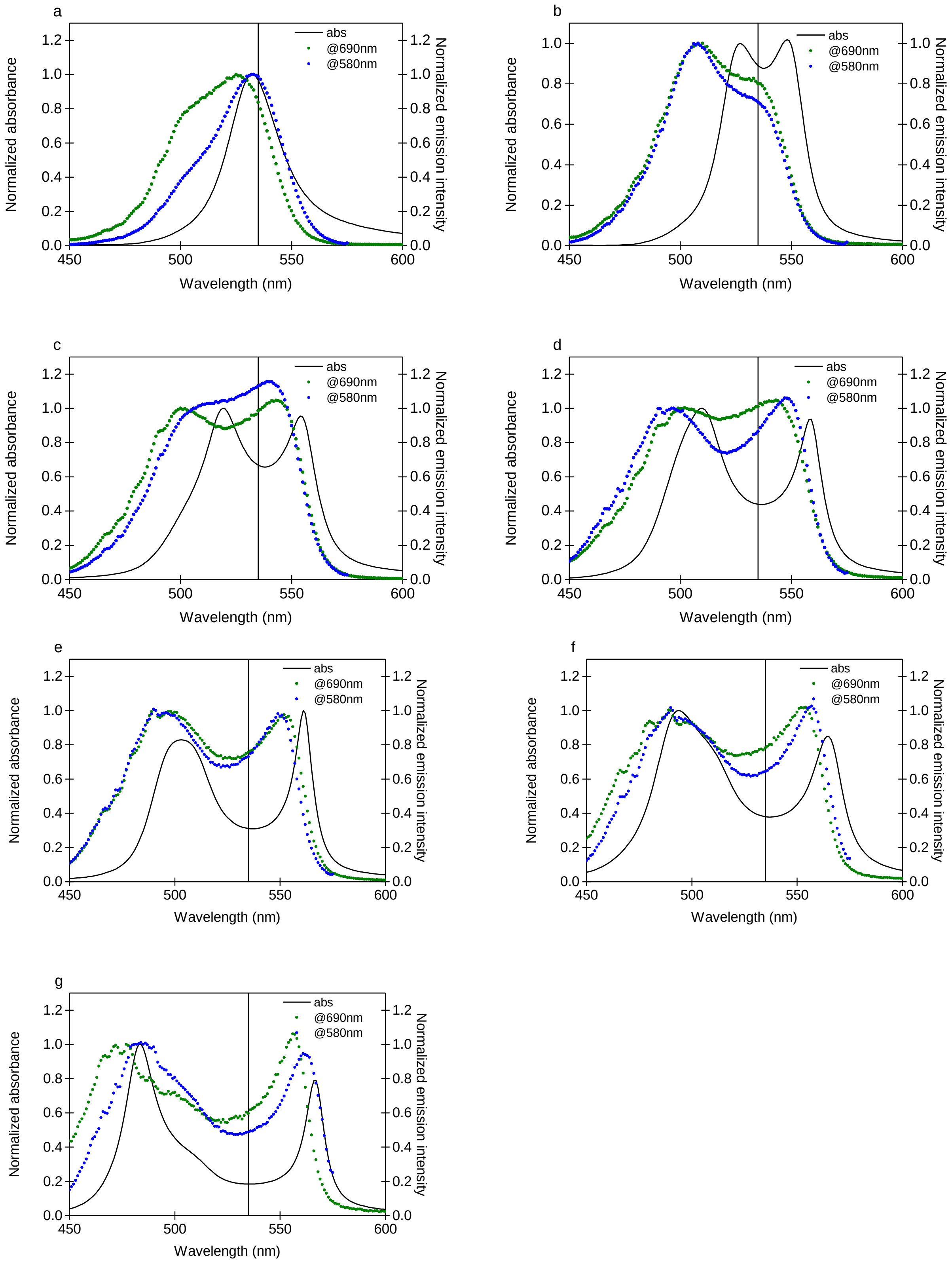

Supplementary Figure 4: Comparison of absorption and excitation spectra. Excitation spectra monitored at 690 (phosphorescence; green dots) and 580 nm (fluorescence; blue dots) of a) 0.01, b) 0.1, c) 0.25, d) 0.30, e) 0.38, f) 0.45 and g) 0.54 M ErB/PVA films inside a cavity compared to cavity absorbance spectra (black line).

Supplementary Note 2: Blue-shift in excitation spectra.

Excitation spectra were found to be slightly blue-shifted relative to the absorbance spectra. The same order of magnitude of blue-shifts has been observed earlier[5-8], and was attributed to the asymmetric absorption band of the state being hybridized.

Supplementary Note 3: Singlet-triplet energy gap of Erythrosine B

The earlier reported energy gap for ErB in PVA matrix (426 meV) was obtained from the spectral separation of the maxima of the fluorescence and phosphorescence[9]. However, Lettinga *et al.* also observed a 3 nm (12 meV) red-shift between fluorescence and delayed fluorescence. By taking this shift in account, the energy gap obtained in this study is in agreement with the earlier reported value.

Supplementary Note 4: Choosing the fitting model for phosphorescence decay.

Since the phosphorescence decays of ErB are non-exponential in a solid matrix, a stretched exponential model is commonly used to analyse the decays[10,11]. In addition, aggregation of the ErB at high concentration adds to the complexity of the decays[12].

Supplementary Table 2: Phosphorescence decay fitting results at different concentrations. Stretched exponents (β), lifetimes (τ) and calculated average phosphorescence lifetimes (τ_T^{avg}) obtained from stretched-exponential tail-fittings of phosphorescence decays at 690 nm for ErB outside and inside a cavity at different concentrations of ErB in PVA. Statistical error for average time constant is calculated using propagation of uncertainties. The statistical errors for τ and β are coming from the fitting.

[ErB] (M)		β	Δβ	τ (μs)	Δτ (μs)	τ_T^{avg} (μs)	$\Delta\tau_T^{avg}$ (μs)
0.02	outside	1.00	0.01	825	65	825	65
	inside	0.74	0.36	679	27	821	438
0.27	outside	0.69	0.07	560	41	718	75
	inside	0.36	0.07	78	34	375	174
0.36	outside	0.61	0.06	451	34	670	72
	inside	0.32	0.05	54	22	365	153
0.45	outside	0.61	0.05	374	26	547	53
	inside	0.41	0.03	87	13	278	45
0.54	outside	0.56	0.03	240	16	397	32
	inside	0.33	0.03	20	5	126	33
0.61	outside	0.55	0.03	200	14	337	28
	inside	0.31	0.02	11	4	91	29

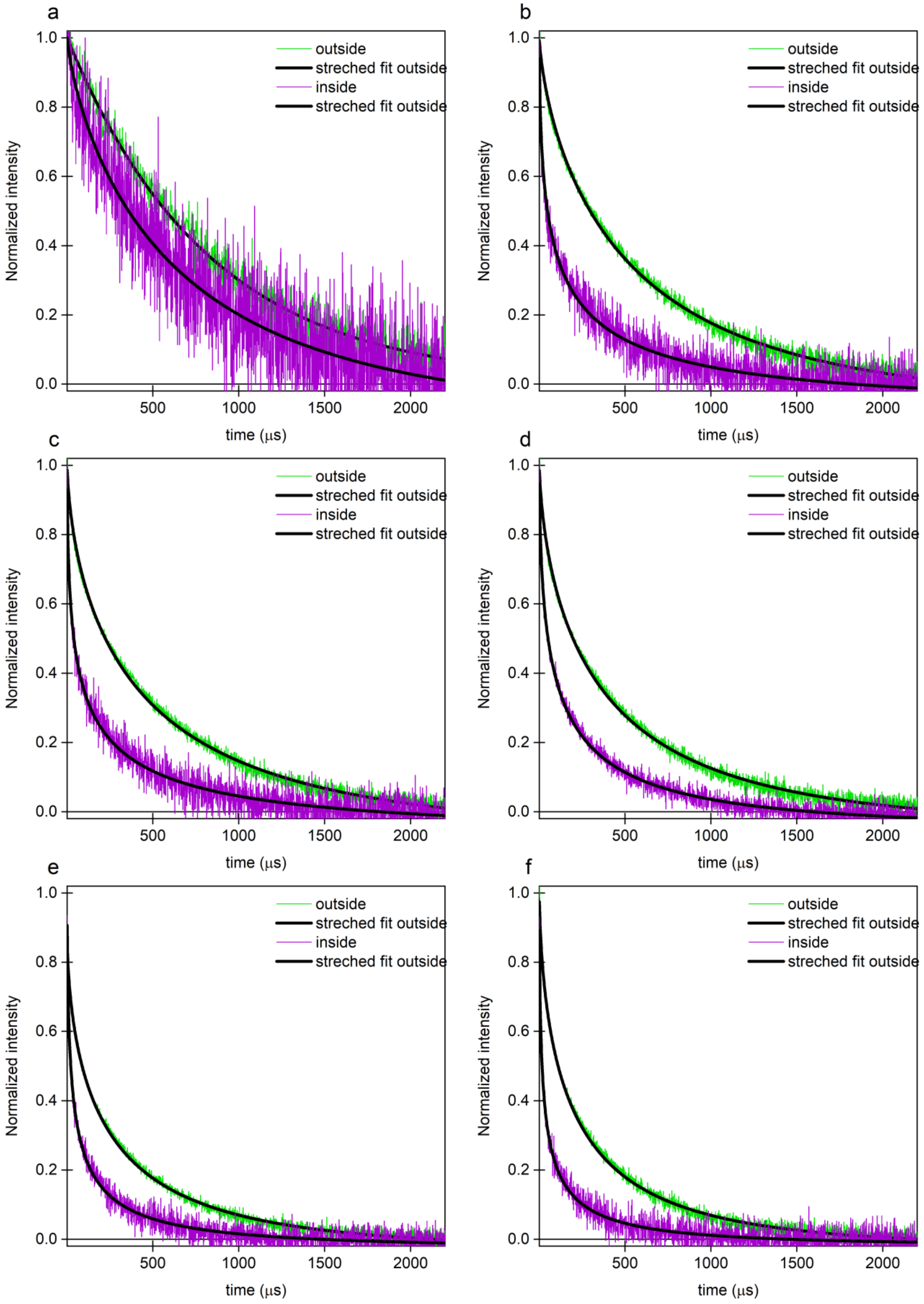

Supplementary Figure 5: Phosphorescence decays at different concentrations. Normalized phosphorescence decay curves and stretched-exponential tail-fittings for a) 0.01, b) 0.27 c) 0.36, d) 0.45, e) 0.54 and f) 0.61 M ErB/PVA films outside and inside a cavity. Samples were excited at maximum of the P+ absorbance and monitored at 690 nm.

Supplementary Note 5: Concentration quenching of Erythrosine B and off-tuning the cavity.

At high concentrations of ErB, also a small decrease in phosphorescence lifetime outside the cavity is seen. Previous studies on ErB have similarly reported triplet decay times to decrease with the increasing concentration due to aggregation[3]. Therefore, the phosphorescence lifetimes inside the cavity were compared to reference samples outside of the cavity to rule out the concentration effect (see Supplementary Table 2). In addition, we noticed that by off-tuning the cavity to red, the lifetime of phosphorescence ($\Delta\tau_T^{avg}$=427 µs) is similar to the phosphorescence lifetime outside the cavity ($\Delta\tau_T^{avg}$=421 µs). The reason for this is because the excitonic contribution to P- was increased when off-tuning the cavity (Supplementary Fig. 6).

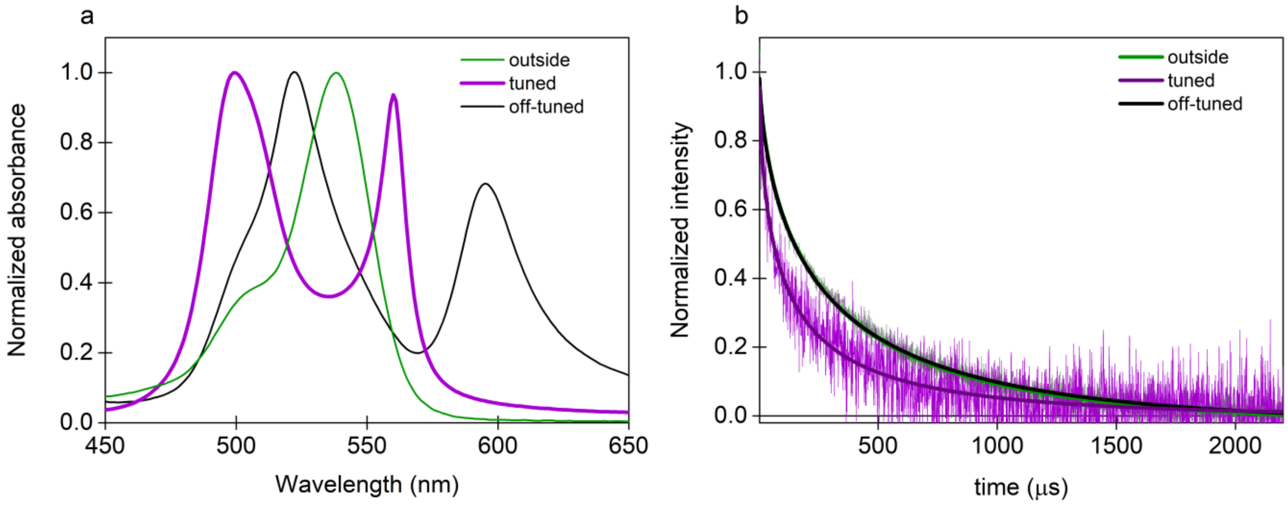

Supplementary Figure 6: Control studies for off-tuned cavity. a) Absorbance spectra of 0.46 M ErB/PVA films outside the cavity (green) and inside the cavity that has been tuned (purple) and off-tuned (black) to resonance. b) Normalized phosphorescence decay curves and stretched-exponential tail-fittings for same samples. Samples were excited at 500 nm (at P^+) and monitored at 690 nm.

Supplementary Table 3: Fitting the change in k_T as a function of singlet-triplet energy gap. Obtained fitting parameters from Eq.3 used in Fig.4b.

k_P+k_{NR} (s^{-1})	k_{ISC} (s^{-1})	T (K)
1215	4.6e8	298 (fixed)

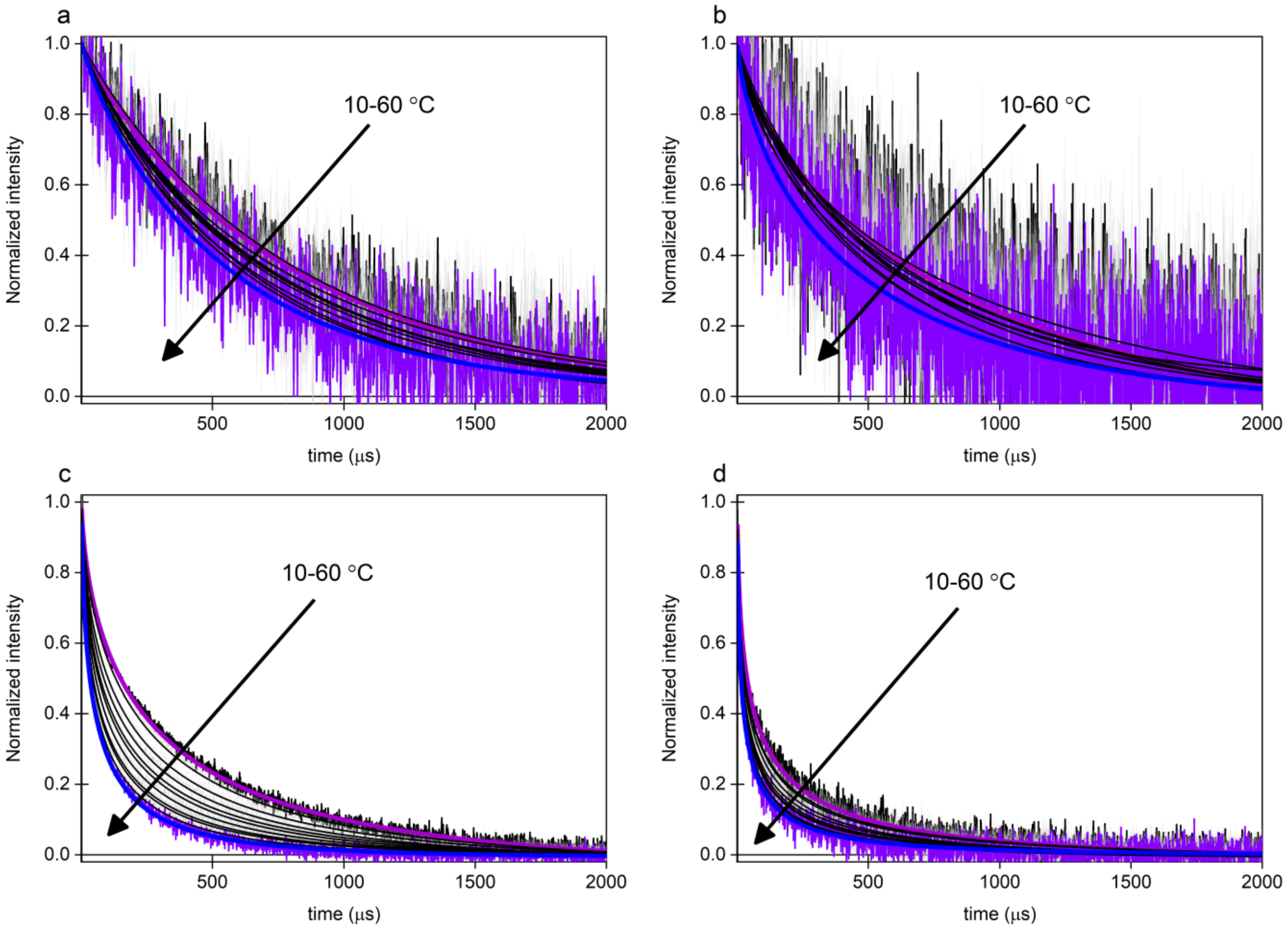

Supplementary Figure 7: Phosphorescence decays at different temperatures. Normalized phosphorescence decay curves and stretched-exponential tail-fittings for ErB/PVA films a) 0.01 M outside, b) 0.01 M inside, c) 0.54 M outside and d) 0.54 M a cavity over the temperature range from 10 to 60 °C. Samples were excited at maximum of the P+ absorbance and monitored at 690 nm.

Supplementary Table 4: Phosphorescence decays fitting results at different temperatures for low concentration samples. Stretched exponents (β), lifetimes (τ) and calculated average phosphorescence lifetimes (τ_T^{avg}) obtained from stretched-exponential tail-fittings of phosphorescence decays at 690 nm for 0.01M ErB ErB/PVA films outside and inside a cavity at different temperatures. Statistical error for average time constant is calculated using propagation of uncertainties. The statistical errors for τ and β are coming from the fitting.

T (°C)	outside						inside					
	β	Δβ	τ (μs)	Δτ (μs)	τ_T^{avg} (μs)	$\Delta\tau_T^{avg}$ (μs)	β	Δβ	τ (μs)	Δτ (μs)	τ_T^{avg} (μs)	$\Delta\tau_T^{avg}$ (μs)
10	0.92	0.25	798	156	828	220	0.83	0.59	751	396	828	595
15	0.96	0.28	790	164	802	226	0.81	0.51	677	342	761	511
20	0.88	0.30	746	188	798	272	0.87	0.59	802	397	859	581
25	0.88	0.31	707	190	755	271	0.88	0.52	719	308	766	448
30	0.91	0.31	710	184	744	256	0.99	0.19	719	257	719	270
35	0.90	0.30	681	176	719	247	0.87	0.45	676	266	723	379
40	0.91	0.29	679	165	708	228	0.76	0.40	551	269	651	398
45	0.86	0.30	629	178	677	251	0.88	0.53	636	317	678	437
50	0.93	0.30	633	157	653	213	0.80	0.50	577	326	653	466
55	0.91	0.28	603	154	630	206	0.80	0.46	532	287	602	404
60	0.89	0.30	544	152	577	207	0.81	0.52	535	310	600	438

Supplementary Table 5: Phosphorescence decays fitting results at different temperatures for high concentration samples. Stretched exponents (β), lifetimes (τ) and calculated average phosphorescence lifetimes (τ_T^{avg}) obtained from stretched-exponential tail-fittings of phosphorescence decays at 690 nm for 0.54M ErB ErB/PVA films outside and inside a cavity at different temperatures. Statistical error for average time constant is calculated using propagation of uncertainties. The statistical errors for τ and β are coming from the fitting.

T (°C)	outside						inside					
	β	Δβ	τ (μs)	Δτ (μs)	τ_T^{avg} (μs)	$\Delta\tau_T^{avg}$ (μs)	β	Δβ	τ (μs)	Δτ (μs)	τ_T^{avg} (μs)	$\Delta\tau_T^{avg}$ (μs)
10	0.57	0.05	288	23	471	48	0.38	0.03	50	12	189	47
15	0.56	0.04	240	19	394	38	0.34	0.03	32	9	174	52
20	0.53	0.03	186	15	337	31	0.36	0.03	33	9	155	44
25	0.51	0.03	153	12	294	26	0.35	0.03	27	7	138	38
30	0.49	0.02	124	10	253	23	0.30	0.02	15	5	138	49
35	0.49	0.02	112	9	232	21	0.32	0.02	16	5	110	34
40	0.47	0.02	89	7	198	17	0.32	0.02	14	4	101	32
45	0.45	0.02	69	6	167	15	0.32	0.02	14	4	94	30
50	0.46	0.02	64	6	153	14	0.31	0.02	10	3	79	26
55	0.45	0.02	53	5	133	12	0.34	0.02	13	4	77	23
60	0.46	0.02	46	4	109	10	0.33	0.02	9	3	60	19

Supplementary Table 6: Fitting the change in k_T as a function of temperature. Fitting parameters for Eq.3 used in Fig.4c-d. The literature value[9] for k_{ISC} is 1.11e9 s^{-1}. Since the triplet state was not coupled, the decay processes from the triplet state to the ground state were considered to stay constant inside the cavity. For high concentration sample, the fitting was thus done by fixing the k_P+k_{NR} to value obtained from low concentration sample fit. Due to aggregation at high concentration, the value of k_{ISC} is known to be slightly lower as compared to value at low concentration[2].

[ErB] (M)		k_P+k_{NR} (s^{-1})	k_{ISC} (s^{-1})	ΔE_{TS} (meV)
0.02	outside	1206	1.5e9	426 (fixed)
	inside	1216	1.4e9	426 (fixed)
0.54	outside	1200 (fixed)	5.7e8	322
	inside	1200 (fixed)	1.7e8	204

Supplementary Table 7: Changes in steady-state absorption and emission spectra due to strong-coupling of samples used for phosphorescence decay measurements. The S_1 absorption (λ_{S1}^{abs}) and fluorescence (λ_{S1}^{emis}) outside a cavity, P- absorption (λ_{P1}^{abs}) and fluorescence (λ_{P1}^{emis}) inside a cavity, and phosphorescence maxima (λ_{T1}^{emis}) outside and inside a cavity at different concentrations of ErB in PVA extracted from spectra in Supplementary Figure 8 and 9.

[ErB]		$\lambda_{S1/P1}^{abs}$, nm	$\lambda_{S1/P1}^{emis}$, nm	λ_{T1}^{emis}, nm
0.02	outside	538	554	691
	inside	538	549	679
0.27	outside	538	562	692
	inside	555	560	684
0.36	outside	538	563	693
	inside	558	562	684
0.45	outside	538	565	694
	inside	561	564	684
0.54	outside	538	566	696
	inside	567	568	685
0.61	outside	538	567	694
	inside	569	569	684

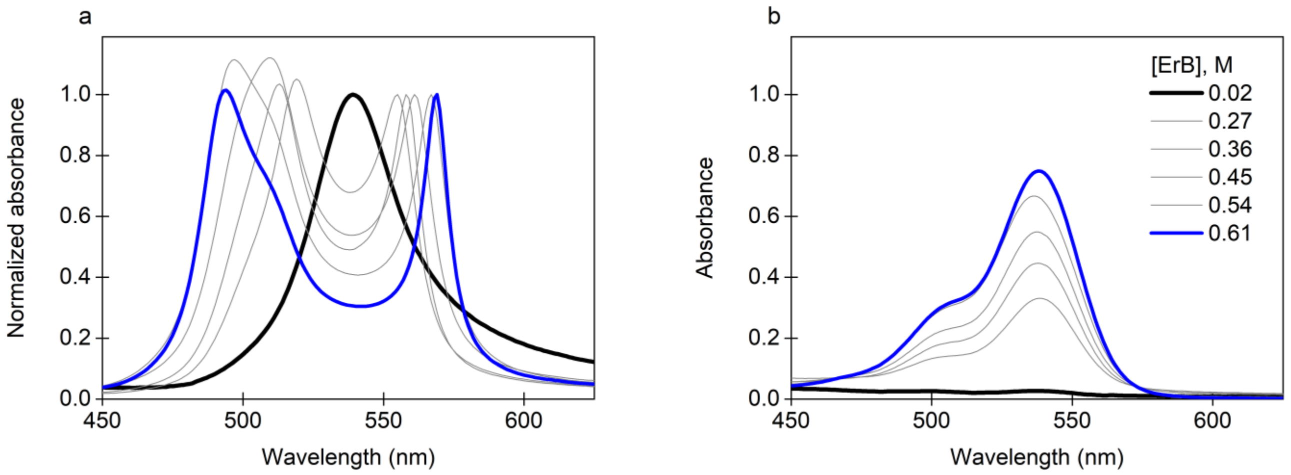

Supplementary Figure 8: Characterization of hybrid light–matter states for new sets of samples used for phosphorescence decay measurements. a) Absorbance spectra of ErB/PVA films inside a cavity that have an ErB concentration between 0.02 M (black) and 0.61 M (blue). b) Absorbance spectra of reference samples for cavities lacking the 18 nm Ag mirror. Concentration of ErB inside the cavity was calculated by using: [ErB] =Abs@538 nm/(82500 M^{-1} cm^{-1} × 150 nm).

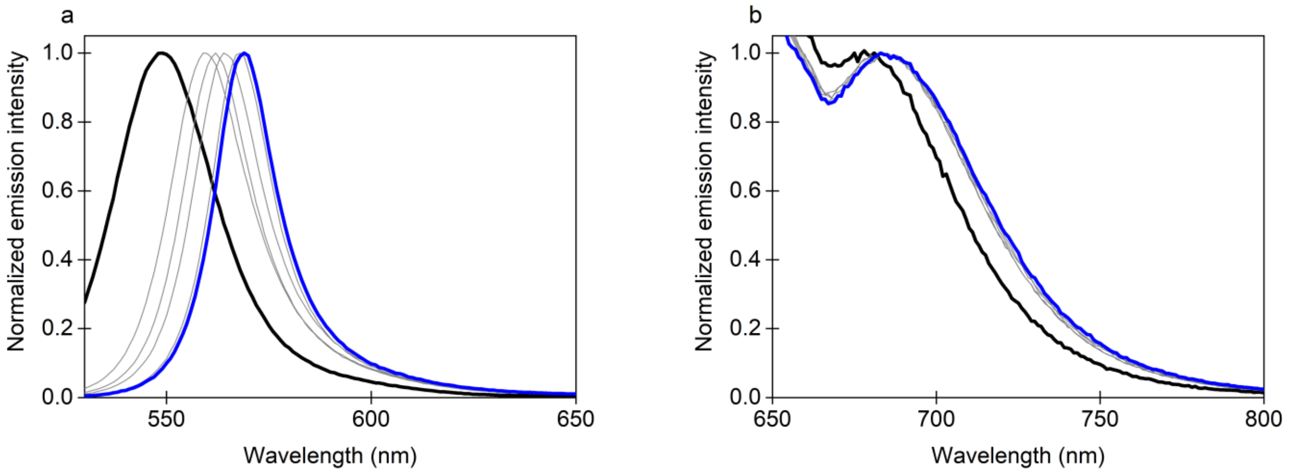

Supplementary Figure 9: Selective coupling of excited states and decrease of triplet-singlet energy gap for new sets of samples used for phosphorescence decay measurements. a) Fluorescence and b) phosphorescence spectra of ErB/PVA films inside a cavity with an ErB concentration between 0.02 (black) and 0.61 M (blue), excited at the maximum of the P+ absorbance.

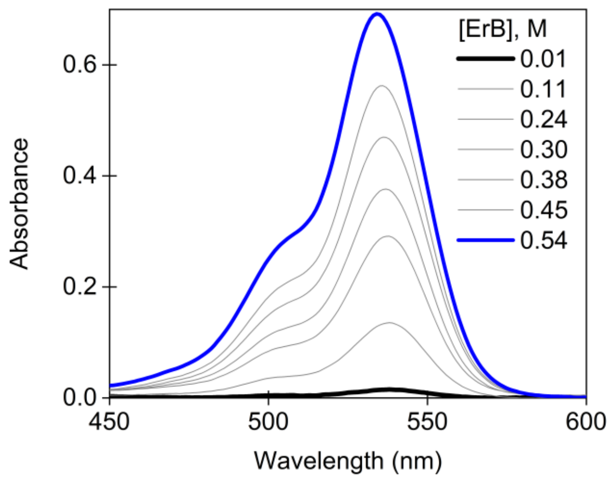

Supplementary Figure 10: Absorbance of reference samples used to determine the concentration inside the cavities. Absorbance spectra of reference samples lacking the 20 nm Ag mirror in Figure 2b. Concentration of ErB inside the cavity was calculated by using: [ErB] =Abs@538 nm/(82500 M^{-1} cm^{-1} × 150 nm).

Supplementary references

1 Byrnes, S. J. Multilayer optical calculations. *arXiv preprint arXiv:1603.02720* (2016).

2 Pant, D. D., Bhagchandani, C. L., Pant, K. C. & Verma, S. P. Aggregation in xanthene dyes, exciton emission and phosphorescence enhancement. *Chem. Phys. Lett.* **9**, 546-547 (1971).

3 Joshi, N. B. & Pant, D. D. Effect of aggregation on the radiative (T1 --> S0) and nonradiative (T1 --> S0 and S1 --> T1) transitions in xanthene dyes. *J. Lumin.* **14**, 1-8 (1976).

4 Stomphorst, R. G. *et al.* Spectroscopic study of erythrosin B in PVA films. *J. Phys. Chem. A* **105**, 4235-4240 (2001).

5 Armitage, A. *et al.* Modelling of asymmetric excitons in organic microcavities. *Synth. Met.* **111**, 377-379 (2000).

6 Coles, D. M. *et al.* Vibrationally Assisted Polariton-Relaxation Processes in Strongly Coupled Organic-Semiconductor Microcavities. *Adv. Funct. Mater.* **21**, 3691-3696 (2011).

7 Coles, D. M. *et al.* Strong coupling between chlorosomes of photosynthetic bacteria and a confined optical cavity mode. *Nat. Commun .* **5**, 5561 (2014).

8 George, J. *et al.* Ultra-strong coupling of molecular materials: spectroscopy and dynamics. *Faraday Discuss.* **178**, 281-294 (2015).

9 Lettinga, M. P., Zuilhof, H. & van Zandvoort, M. A. M. J. Phosphorescence and fluorescence characterization of fluorescein derivatives immobilized in various polymer matrices. *Phys. Chem. Chem. Phys.* **2**, 3697-3707 (2000).

10 Pravinata, L. C., You, Y. & Ludescher, R. D. Erythrosin B Phosphorescence Monitors Molecular Mobility and Dynamic Site Heterogeneity in Amorphous Sucrose. *Biophys. J.* **88**, 3551-3561 (2005).

11 You, Y. & Ludescher, R. D. Phosphorescence of Erythrosin B as a Robust Probe of Molecular Mobility in Amorphous Solid Sucrose. *Appl. Spectrosc.* **60**, 813-819 (2006).

12 Dias, F. B., Penfold, T. J. & Monkman, A. P. Photophysics of thermally activated delayed fluorescence molecules. *Methods Appl Fluoresc* **5**, 012001 (2017).
